# Case Report: Endodontic management of a fused maxillary lateral incisor and canine with split

**DOI:** 10.3389/fdmed.2026.1954240

**Published:** 2026-09-23

**Authors:** Alen Romel, Adithya Kashyap K, S. Vidhyadhara Shetty, K. Harish Shetty, Nikhil Harikrishnan

**Affiliations:** 1 Department of Conservative Dentistry & Endodontics, Yenepoya Dental College, Yenepoya (Deemed to be University), Mangaluru, Karnataka, India; 2 Department of Conservative Dentistry & Endodontics, KVG Dental College & Hospital, Sullia, Karnataka, India

**Keywords:** cone-beam computed tomography, crowns, endodontics, fused teeth, root canal treatment, tooth anomalies

## Abstract

**Background:**

Dental fusion, characterized by the joining of two neighboring tooth germs during odontogenesis, is an uncommon condition affecting approximately 0.1% of the permanent dentition. The unusual anatomy of the pulp chamber and the unpredictable root canal system pose significant challenges during endodontic treatment.

**Case report:**

A 47-year-old female patient presented with a 6-month history of spontaneous nocturnal pain in the upper left anterior region. Clinical examination revealed a solitary tooth occupying the positions of teeth 22 and 23; the tooth had a buccal groove and severe distoproximal decay, with no tooth 23. Two pulp chambers joining into one canal in the middle third of the root and a distal periapical radiolucency were found on periapical radiography and cone beam computed tomography imaging.

**Management:**

Root canal treatment was performed using nickel–titanium rotary instrumentation with activated irrigation and warm vertical obturation, followed by a split porcelain-fused-metal crown.

**Conclusion:**

At the 6-month follow-up, the tooth was asymptomatic tooth with improving periapical condition, thus proving that the interdisciplinary approach guided by imaging provides predictable results in the treatment of permanently fused teeth.

## Introduction

Dental fusion is a congenital anomaly marked by the fusion of two separate tooth buds in the process of odontogenesis, resulting in the formation of one enlarged tooth (1). Fusion can either be complete, in which both the crown and root are involved, or partial, depending on the time when fusion takes place during development (2). The tooth has characteristics of crown widening mesiodistally, unusual root and root canal morphology, and is usually associated with loss of tooth on the opposite side of the arch (3).

The prevalence of dental fusion in different populations has been reported to vary between 0.08% and 3.0% for all teeth, 0.5%–2.5% for primary teeth, and 0.1% for permanent teeth (4). Population-based data from India have shown similar prevalence, with values of 1%–1.5% for the primary dentition (5). Dental fusion occurs mostly in the anterior region and can be clinically diagnosed based on a broad buccolingual or mesiodistal crown, the presence of a groove along the labial/lingual surface of the tooth, a reduction in the number of teeth in the arch, and complexity in the internal structure as seen on radiographs (3). Among the permanent dentition, fusion of the maxillary lateral incisor with the canine is exceptionally rare (6).

The management of endodontically fused teeth poses great challenges. Depending on the pulp chamber arrangement, it could be a case of two individual pulp chambers, bifurcated canals, or a fused chamber that requires a specific technique to access, prepare cavities, negotiate, and fill the canal (7). Lack of awareness of the real nature of the root canal system makes it easy to make errors such as incomplete debridement, persistent infections, strip perforation, and even fracture of instruments (8). The introduction of cone beam computed tomography has greatly helped the practitioner understand root canal morphology before instrumentation begins (9). Recent clinical reports have further emphasized that CBCT-guided treatment planning is particularly useful when fused teeth demonstrate multiple canals or canal convergence, allowing the clinician to determine the number, course, and point of union of the canals before instrumentation (10–12).

Following endodontics, the unique crown morphology of fused teeth must be accounted for during prosthetic procedures. The increased mesiodistal diameter, irregular crown shape, and fusion groove pose challenges during preparation and design of crowns. Splitting of crowns and the use of porcelain-fused-to-metal (PFM) restorations have proved effective in managing such the fused teeth.

The present report describes the diagnosis, endodontic management, and prosthetic rehabilitation of a fused maxillary lateral incisor and canine (teeth 22 and 23) in an adult patient with follow-up for 6 months through clinical and radiographic examinations. This case report aims to emphasize the importance of an interdisciplinary approach and the effectiveness of modern procedures.

## Case report

A 47-year-old woman presented to the Department of Conservative Dentistry and Endodontics with a primary complaint of pain in the area of the upper left anterior teeth lasting for 6 months. It was spontaneous and intermittent pain that occurred mostly at night and was partially alleviated by analgesics. The patient had no relevant systemic diseases, significant dental history, familial dental anomalies, or social history.

Intraorally, only one large tooth was found in the area of the 22 and 23 teeth, having a bulbous crown buccolingually and an increased mesiodistal width (Figure 1A). Deep distoproximal caries with subgingival extension was diagnosed, and a pronounced vertical groove was noted on the buccal aspect, suggesting fusion of the lateral incisor and canine. Tooth 23 was clinically absent as an independent tooth because it was fused with tooth 22, resulting in a reduced tooth count in the affected region and supporting the diagnosis of true fusion. There was tenderness on percussion. Sensibility testing of the fused tooth demonstrated a prolonged response to cold and electric pulp stimuli. These findings, together with the history of spontaneous nocturnal pain, were consistent with symptomatic irreversible pulpitis. However, because the fused tooth contained two distinct pulp chambers that subsequently converged into a single canal, chamber-specific pulpal status could not be definitively established. The periodontium was unremarkable clinically, without pathological probing depths.

**Figure 1:**
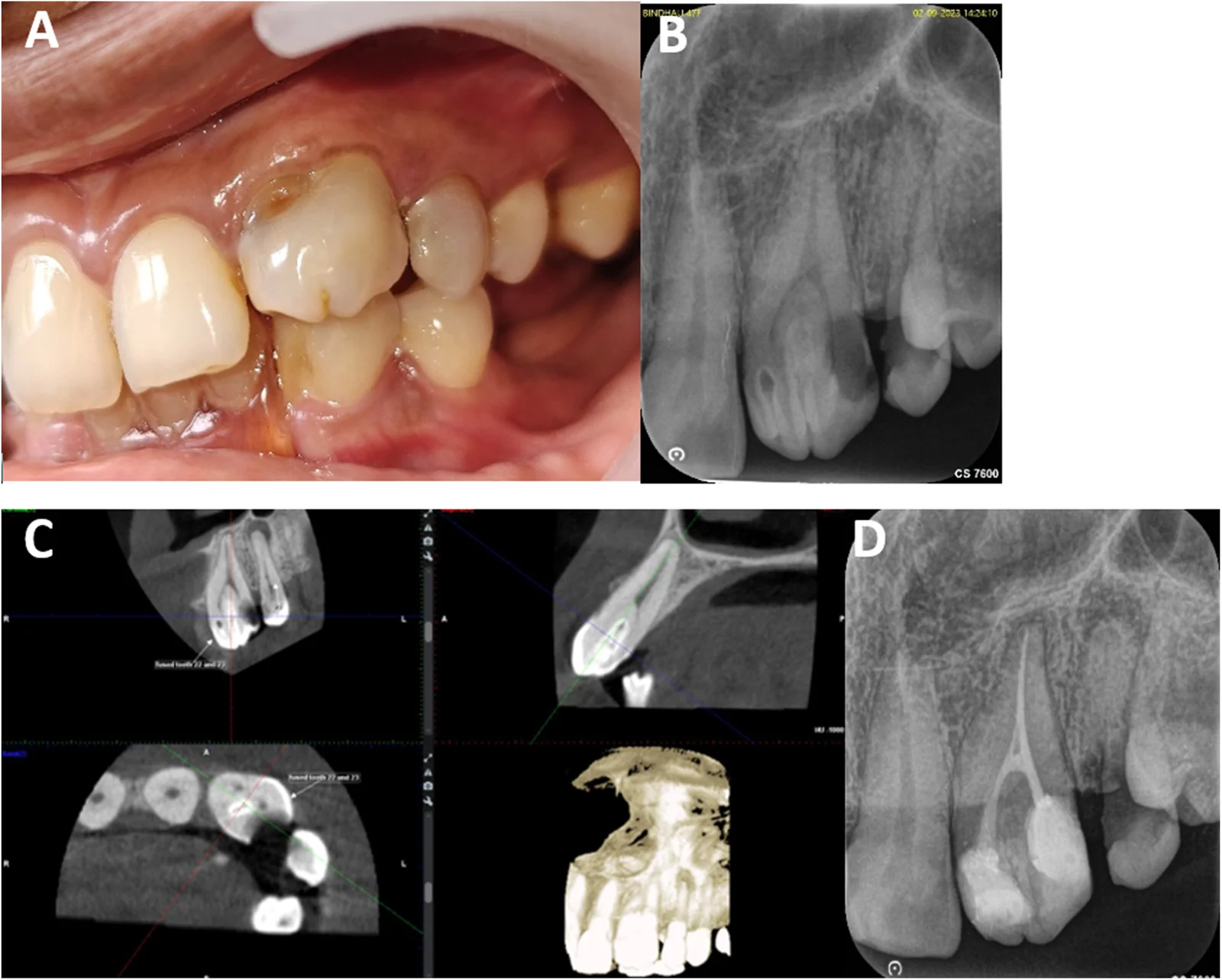
Preoperative and intraoperative findings for the fused 22–23 unit. **(A)** Clinical photograph showing the bulbous crown, increased mesiodistal width, buccal fusion groove, disto-proximal caries and absent tooth 23. **(B)** Periapical radiograph showing mesial and distal pulp chambers converging into a single canal, with distal periapical radiolucency. **(C)** Cone-beam computed tomography confirming canal fusion at the middle third of the root. **(D)** Postoperative radiograph after obturation, confirming satisfactory fill of both canals.

On the periapical radiography, there was only one enlarged tooth found between teeth 22 and 23 (Figure 1B). There were two separate pulp chambers, namely, the mesial and distal chambers, which were distinguished by deep vertical invagination, and converged into one root canal in the apical part of the tooth. In the distal area, a large periapical radiolucency that probably originated from the distal pulp chamber. With the help of cone beam computed tomography (CBCT), there was established three-dimensional root canal anatomy (Figure 1C). It was found out that the mesial and distal canals united in the middle third of the root and formed one apical canal. This information was crucial for understanding canal configuration, planning access and instrumentation, and anticipating canal convergence, which could not be adequately appreciated on two-dimensional imaging alone.

The final diagnosis was fusion of maxillary teeth 22 and 23, with a clinical pulpal diagnosis of symptomatic irreversible pulpitis based on sensibility testing and a periapical diagnosis of symptomatic apical periodontitis. The differential diagnosis included gemination, dens invaginatus, macrodontia, and concrescence. The non-appearance of tooth 23 in the dentition, the presence of two different pulp chambers, and CBCT proving fusion of the canals confirmed the diagnosis of dental fusion. The treatment plan comprised three main phases, i.e., prophylaxis, endodontic treatment, and restoration with a PFM split crown.

Following oral prophylaxis, endodontic treatment was initiated under local anesthesia with rubber dam isolation. The access cavity outline was modified to account for the fused morphology and the presence of two separate pulp chambers, incorporating a broader design to permit identification and negotiation of both the mesial and distal orifices. Working length was determined using an electronic apex locator in conjunction with periapical radiographic verification. Instrumentation was performed using HyFlex EDM nickel–titanium rotary files (Coltène/Whaledent AG, Altstätten, Switzerland) in a crown-down sequence with 20/0.04, 25/0.04, and 35/0.04 instruments. The final apical preparation of the merged canal was completed to size 35/0.04 at a rotational speed of 400 rpm and a torque setting of 2.5 N·cm to the established working length. Irrigation was performed using 3% sodium hypochlorite alternated with 17% ethylenediaminetetraacetic acid for smear layer removal. During the final irrigation flush, sonic activation was performed using the EndoActivator system (Dentsply Sirona, Tulsa, OK, USA) to enhance irrigant penetration into the anatomical irregularities of the fused root canal system. The irrigant was activated with the appropriate EndoActivator polymer tip placed within the canal without binding, using the manufacturer’s recommended operating settings and activation protocol. Following drying with sterile paper points, a size 35/0.04 master gutta-percha cone was fitted to the established working length and verified radiographically. The canal was subsequently obturated using warm vertical compaction with gutta-percha and a bioceramic-based sealer (Figure 1D). The access cavity was restored with a resin composite restorative material (Tetric N-Ceram, Ivoclar, Schaan, Liechtenstein) and maintained until definitive prosthetic rehabilitation.

Following an uneventful 2-week review, prosthetic rehabilitation of the fused unit was undertaken. Given the anomalous mesiodistal dimensions and the presence of the fusion groove, a split PFM crown design was selected. The fused tooth was prepared as two distinct crown units along the line of the fusion groove, respecting the individual boundaries of the lateral incisor and canine components while ensuring adequate structural support for the restored unit. Provisionalization provided uniform preparation depth, an ideal taper, and a definite finish line. The procedure helped maintain the emergence form of all units and provided for better aesthetic and occlusal integration than a single large crown. Impressions were made with addition silicone impression material and sent to the lab for fabrication of the split PFM crowns, which were then definitively cemented with glass ionomer luting cement after ensuring fit, esthetics, and occlusion (Figure 2A).

**Figure 2:**
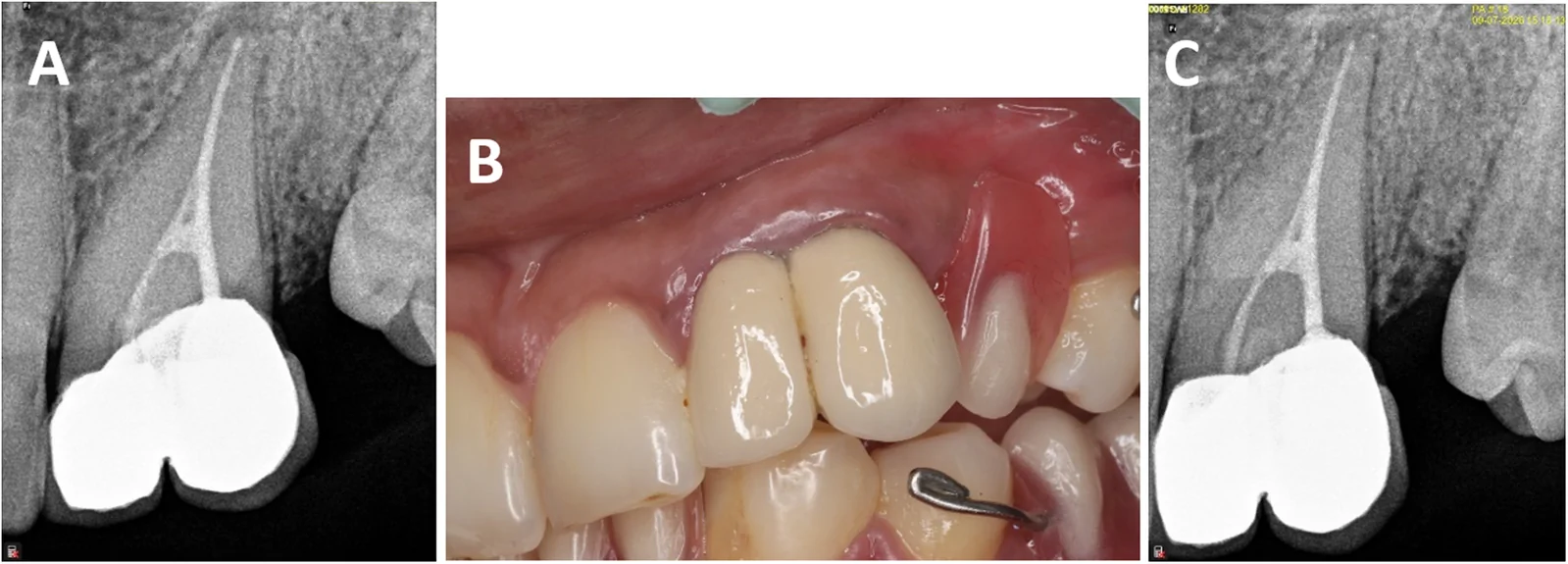
Prosthetic rehabilitation and 6-month follow-up. **(A)** Periapical radiograph after cementation of the split porcelain-fused-to-metal crown **(B)** Six-month clinical photograph showing an intact restoration and healthy periodontal tissues. **(C)** Six-month periapical radiograph showing progressive resolution of the periapical radiolucency and periapical bone regeneration.

The patient was reviewed at 1 week, 1 month, 3 months, and 6 months after obturation. At the 6-month appointment, the patient was completely asymptomatic, reporting no spontaneous pain, sensitivity to percussion or palpation, or no soft tissue swelling or sinus tract formation. The split PFM crown was clinically intact, with satisfactory esthetics, occlusal contacts, and gingival health (Figure 2B). Periapical radiography at 6 months demonstrated a reduction in the extent and radiolucency of the preexisting periapical lesion, with increased radiopacity and evidence of osseous fill in the periapical region (Figure 2C). Radiographic assessment of periapical healing was based on a qualitative comparison of the preoperative and 6-month periapical radiographs. The assessment considered the change in the extent and radiolucency of the pre-existing periapical lesion, the definition of its borders, and evidence of increased radiopacity consistent with osseous fill. Based on these findings, the lesion was considered to show incomplete periapical healing at 6 months. No signs of root resorption, instrument separation, or procedural errors were identified, and the prognosis for the treated unit was considered favorable, with long-term monitoring recommended at annual intervals. The timeline of presentation, investigations, diagnosis, treatment, and follow-up is presented in Table 1.

**Table 1:** CARE-compliant timeline of presentation, investigations, diagnosis, treatment and follow-up for the fused 22–23 unit.

Timeline	
Time point	Event/finding
Presentation (Day 0)	47-year-old woman presented with a 6-month history of spontaneous, intermittent nocturnal pain in the upper left anterior region, partially relieved by analgesics
Day 0—Investigations	Clinical examination, periapical radiography and CBCT: single fused tooth in the 22–23 region, two pulp chambers converging into one canal, distal periapical radiolucency, canal fusion confirmed at the middle third of the root
Day 0—Diagnosis	The final diagnosis was fusion of maxillary teeth 22 and 23, with a clinical pulpal diagnosis of symptomatic irreversible pulpitis based on sensibility testing and a periapical diagnosis of symptomatic apical periodontitis
Day 0—Treatment visit 1	Oral prophylaxis followed by root canal treatment under rubber dam isolation: modified access, HyFlex EDM instrumentation, NaOCl/EDTA irrigation with activation, warm vertical obturation with bioceramic sealer; access cavity temporized
2 weeks	Review visit: uneventful healing. Tooth prepared as two crown units along the fusion groove; impressions made for a split PFM crown
Treatment visit 2	Split porcelain-fused-to-metal crown cemented with glass-ionomer luting cement after verification of fit, aesthetics and occlusion
1 week, 1 month, 3 months postobturation	Routine follow-up visits; no adverse events reported
6 months postobturation (final outcome)	Patient completely asymptomatic; crown clinically intact with healthy periodontal tissues; periapical radiograph showed progressive resolution of the radiolucency with periapical bone regeneration; prognosis favorable, annual monitoring recommended

## Discussion

The present case illustrates the diagnostic and technical complexity inherent to the endodontic and prosthetic management of a fused permanent maxillary tooth, and documents a successful outcome at the 6-month follow-up with the patient remaining completely asymptomatic. Fusion in the permanent dentition carries a prevalence of approximately 0.1%, and the published case series guiding its management are limited, lending additional value to the reporting of well-documented individual cases (4, 6, 11, 12).

The pathognomonic features of a widened crown, a vertical fusion groove, and a reduced arch tooth count were all present in this case (3). Distinguishing fusion from gemination is a common diagnostic challenge: in fusion, the total tooth count is reduced by one, whereas in gemination it remains normal (2, 6). The absence of tooth 23 from the arch was therefore the critical discriminator confirming true fusion. Concrescence, in which two teeth are united only by cementum after root formation, and dens invaginatus were excluded on the basis of the CBCT findings, which demonstrated a shared pulpal anatomy with two chambers converging into a single root canal (10).

The clinical presentation of the present case is broadly consistent with previously reported cases of fused teeth, in which caries or pulpal and periapical diseases may develop due to anatomical irregularities that predispose to plaque accumulation and compromise oral hygiene maintenance (11, 12). In the present patient, the deep distoproximal caries was accompanied by spontaneous nocturnal pain and percussion tenderness, indicating both pulpal and periapical involvement (11). Similar presentations involving pain and periapical pathology have been described in recent reports of fused teeth, although the exact clinical manifestations vary according to the teeth involved and the extent of fusion (12).

CBCT assessment was pivotal. Identification of canal fusion at the middle third of the root—information that was not fully appreciable on two-dimensional periapical radiography—directly informed the access cavity design, canal negotiation and instrumentation strategy, and the anticipation of challenges at the level of canal convergence (8, 9). Contemporary evidence consistently supports preoperative CBCT evaluation in teeth with anomalous morphology, highlighting its role in reducing procedural errors and refining prognostic assessment (9).

The choice of HyFlex EDM nickel–titanium files for canal shaping was guided by their demonstrated cyclic fatigue resistance and controlled memory behavior, which enables safe navigation of irregular and curved canal systems without generating excessive lateral forces (13). Activation of the irrigant during the final rinse promoted penetration into the deep vertical invagination and canal irregularities inherent to the fused morphology, enhancing both debridement and disinfection (13, 14).

Prosthetic management of endodontically treated fused teeth poses another unique set of challenges that have received less attention than deserved. An oversized crown covering the whole mesiodistal dimension of the fused tooth will most likely result in an unsatisfactory emergence profile, compromised proper oral hygiene maintenance, gingival irritation, and an esthetically unacceptable outcome (15). To overcome these shortcomings, the split PFM crown approach was chosen because of respect for the natural borders of the tooth along the fusion line and the possibility to independently restore the lateral incisor and canine components, thereby providing a more anatomical emergence profile and superior esthetics. The choice of PFM crowns was dictated by their proven longevity and ability to provide structural integrity in the face of the modified crown preparation geometry (16). In cases where esthetics is the primary concern, an all-ceramic split crown can be used in the future.

The 6-month follow-up demonstrated complete resolution of symptoms with radiographic confirmation of periapical bone regeneration. Such a period is typical for periapical tissue healing following root canal therapy; early osseous fill may be seen on periapical radiographs between three and 6 months post-treatment in the presence of a preexisting lesion (17). While 6 months is a significant milestone, further evaluation at 1-, 2-, and 5-year intervals is necessary to assess the long-term sustainability of endodontic and prosthetic treatment outcomes. Given the potential risk of root fracture in teeth with fused roots due to their complex root canal structure and reduced dentin thickness at the level of union, ongoing observation is necessary (7).

This report is subject to the limitations inherent to a single case: its results cannot be generalized, and conclusions regarding the effectiveness of treatment must be confirmed by other studies. The pulpal diagnosis warrants cautious interpretation because sensibility testing was performed at the fused-tooth level, while the tooth exhibited two distinct pulp chambers and a large distal periapical radiolucency; therefore, the pulpal status of each component could not be independently determined. Nevertheless, this case contributes to the growing body of evidence supporting an interdisciplinary, imaging-guided approach to the management of fused permanent teeth.

## Conclusion

This report documents the successful interdisciplinary management of a fused maxillary lateral incisor and canine in a 47-year-old patient, combining endodontic treatment using HyFlex EDM nickel–titanium files and activated irrigation with prosthetic rehabilitation by a split porcelain-fused-to-metal crown. At the 6-month follow-up, the patient was completely asymptomatic, with radiographic evidence of improving periapical status and osseous repair. Meticulous preoperative diagnosis supported by CBCT, careful endodontic management respecting the anomalous canal architecture, and prosthetic planning tailored to the fused morphology were the key determinants of clinical success. Clinicians encountering similar presentations are encouraged to adopt an individualized, evidence-based, and interdisciplinary approach, with continued long-term monitoring.

## Patient perspective

“I initially sought treatment because of persistent discomfort in my tooth and was concerned about the possibility of losing it. However, throughout the treatment process, I was made aware of the treatment procedures and what was happening, which made me feel relaxed. Even though the treatment involved more than one appointment, it was very comfortable, and I experienced an improvement in the symptoms. When I revisited after the treatment process, I found out that the tooth was working perfectly fine without any pain.”

## Data Availability

The original contributions presented in the study are included in the article/Supplementary Material, further inquiries can be directed to the corresponding author.

## References

[1] Ben Salem M Chouchene F Masmoudi F Baaziz A Maatouk F Ghedira H. Fusion or gemination? Diagnosis and management in primary teeth: a report of two cases. Case Rep Dent. (2021) 2021:6661776. 10.1155/2021/6661776 34123435 PMC8189771

[2] Aguiló L Gandia JL Cibrian R Catalá M. Primary double teeth. A retrospective clinical study of their morphological characteristics and associated anomalies. Int J Paediatr Dent. (1999) 9:175–83. 10.1046/j.1365-263x.1999.00131.x 10815574

[3] Demircioglu Guler D Sen Tunc E Arici N Ozkan N. Multidisciplinary management of a fused tooth: a case report. Case Rep Dent. (2013) 2013(1):634052. 24396611 10.1155/2013/634052 PMC3874312

[4] Hagman FT. Anomalies of form and number, fused primary teeth, a correlation of the dentitions. ASDC J Dent Child. (1988) 55(5):359–61. 3049713

[5] Gupta SK Saxena P Jain S Jain D. Prevalence and distribution of selected developmental dental anomalies in an Indian population. J Oral Sci. (2011) 53:231–8. 10.2334/josnusd.53.231 21712629

[6] Grover PS Lorton L. Gemination and twinning in the permanent dentition. Oral Surg Oral Med Oral Pathol. (1985) 59:313–8. 10.1016/0030-4220(85)90173-2 3856826

[7] Sivolella S Bressan E Mirabal V Stellini E Berengo M. Extraoral endodontic treatment, odontotomy and intentional replantation of a double maxillary lateral permanent incisor: case report and 6-year follow-up. Dent Traumatol. (2011) 27:303–8. 10.1111/j.1365-2591.2008.01391.x 18363700

[8] Patel S Durack C Abella F Shemesh H Roig M Lemberg K. Cone beam computed tomography in endodontics–a review. Int Endod J. (2015) 48:3–15. 10.1111/iej.12270 24697513

[9] Estrela C Bueno MR Leles CR Azevedo B Azevedo JR. Accuracy of cone beam computed tomography and panoramic and periapical radiography for detection of apical periodontitis. J Endod. (2008) 34:273–9. 10.1016/j.joen.2007.11.023 18291274

[10] Mei X-H Liu J Wang W Zhang Q-X Hong T Bai S-Z. Endodontic management of a fused left maxillary second molar and two paramolars using cone beam computed tomography: a case report. World J Clin Cases. (2022) 10:8367–74. 10.12998/wjcc.v10.i23.8367 36159529 PMC9403698

[11] Sener HS Celer AM Tumen EC. Conservative management of a fused maxillary molar and supernumerary premolar using CBCT: a case report with 2-year follow-up. Contemp Pediatr. (2025) 6(3):254–62. 10.51463/cpd.2025.43

[12] Isaabadi M Yaghobee S Bolhari B Malekpour F. Multidisciplinary management of a permanent central incisor fused with a supernumerary tooth: a case report. Frontiers in Dentistry. (2025) 22:44. 10.18502/fid.v22i44.19977 41492563 PMC12765353

[13] Pirani C Iacono F Generali L Sassatelli P Nucci C Lusvarghi L. Hyflex EDM: superficial features, metallurgical analysis and fatigue resistance of innovative NiTi rotary instruments. Int Endod J. (2016) 49:483–93. 10.1111/iej.12470 26011181

[14] Van Der Sluis LW Versluis M Wu MK Wesselink PR. Passive ultrasonic irrigation of the root canal: a review of the literature. Int Endod J. (2007) 40:415–26. 10.1111/j.1365-2591.2007.01243.x 17442017

[15] Neves AA Neves ML Farinhas JA. Bilateral connation of permanent mandibular incisors: a case report. Int J Paediatr Dent. (2002) 12:61–5. 10.1046/j.0960-7439.2001.00324.x 11858216

[16] Pjetursson BE Sailer I Makarov NA Zwahlen M Thoma DS. All-ceramic or metal-ceramic tooth-supported fixed dental prostheses (FDPs)? A systematic review of the survival and complication rates. Dent Mater. (2015) 31:624–39. 10.1016/j.dental.2015.02.013 25935732

[17] Ricucci D Siqueira JF Jr. Fate of the tissue in lateral canals and apical ramifications in response to pathologic conditions and treatment procedures. J Endod. (2010) 36:1–15. 10.1016/j.joen.2009.09.038 20003929

